# A Photoactive Supramolecular Complex Targeting PD-L1 Reveals a Weak Correlation between Photoactivation Efficiency and Receptor Expression Levels in Non-Small-Cell Lung Cancer Tumor Models

**DOI:** 10.3390/pharmaceutics15122776

**Published:** 2023-12-14

**Authors:** Pietro Delcanale, Manuela Maria Alampi, Andrea Mussini, Claudia Fumarola, Maricla Galetti, Pier Giorgio Petronini, Cristiano Viappiani, Stefano Bruno, Stefania Abbruzzetti

**Affiliations:** 1 Department of Mathematical, Physical and Computer Sciences, University of Parma, 43124 Parma, Italy; pietro.delcanale@unipr.it (P.D.); manuelamaria.alampi@unipr.it (M.M.A.); andrea.mussini@unipr.it (A.M.); cristiano.viappiani@unipr.it (C.V.); 2 Department of Medicine and Surgery, University of Parma, 43125 Parma, Italy; claudia.fumarola@unipr.it (C.F.); piergiorgio.petronini@unipr.it (P.G.P.); 3 Department of Occupational and Environmental Medicine, Epidemiology and Hygiene, INAIL-Italian Workers’ Compensation Authority, 00078 Rome, Italy; m.galetti@inail.it; 4 Department of Food and Drug, University of Parma, 43124 Parma, Italy; stefano.bruno@unipr.it

**Keywords:** targeted photodynamic therapy, photo-immunotherapy, PD-L1, dSTORM, eosin, photosensitizer

## Abstract

Photo-immunotherapy uses antibodies conjugated to photosensitizers to produce nanostructured constructs endowed with targeting properties and photo-inactivation capabilities towards tumor cells. The superficial receptor density on cancer cells is considered a determining factor for the efficacy of the photodynamic treatment. In this work, we propose the use of a photoactive conjugate that consists of the clinical grade PD-L1-binding monoclonal antibody Atezolizumab, covalently linked to either the well-known photosensitizer eosin or the fluorescent probe Alexa647. Using single-molecule localization microscopy (direct stochastic optical reconstruction microscopy, dSTORM), and an anti-PD-L1 monoclonal antibody labelled with Alexa647, we quantified the density of PD-L1 receptors exposed on the cell surface in two human non-small-cell lung cancer lines (H322 and A549) expressing PD-L1 to a different level. We then investigated if this value correlates with the effectiveness of the photodynamic treatment. The photodynamic treatment of H322 and A549 with the photo-immunoconjugate demonstrated its potential for PDT treatments, but the efficacy did not correlate with the PD-L1 expression levels. Our results provide additional evidence that receptor density does not determine a priori the level of photo-induced cell death.

## 1. Introduction

Photodynamic therapy (PDT) is a cancer treatment that involves the concomitant presence of a photosensitizer (PS), visible light at a specific wavelength for PS activation, and oxygen, which reacts with PS to generate reactive oxygen species (ROS) that in turn lead to cell death [1,2].

PDT has remarkable advantages over conventional treatments. Since it relies on the illumination of the tissues of interest, it is more spatially selective and thus less toxic than chemotherapeutic drugs. Moreover, PDT exploits photo-oxidative damages that hit a variety of molecular targets. As such, it is less prone to induce resistance [3,4]. Besides direct tumor cell death, damage to the microvasculature and the induction of a local inflammatory reaction represent important adjuvant mechanisms in cancer treatment. The local inflammatory response leads to neutrophil and inflammatory cell accumulation at the treated sites. The inflammatory response slowly develops into adaptive immunity followed by systemic immunity induction [5]. In spite of the relatively large number of compounds with potential for application in PDT, only a handful are currently approved for clinical applications [6,7].

A lack of tumor selectivity and poor tumor accumulation are still among the major existing limitations of PDT. This usually results in side effects that include skin photosensitivity and the destruction of healthy tissue. To address these issues, current efforts aim to develop PSs targeted to molecular species overexpressed in tumors, either by directly conjugating the PS to a ligand with high affinity for a specific cellular component, or by incorporating the PS into a multifunctional nanostructure with targeting capability and diagnostic potential [4,8,9,10,11,12,13]. In particular, an interesting strategy consists in using monoclonal antibodies as targeting carriers, exploiting their capability to address tumor cells overexpressing antigens on the plasma membrane [14]. This technique, termed photo-immunotherapy [15], has been demonstrated to minimize effects on surrounding normal cells [16] and to be effective with different antigen/antibody combinations, such as CD20 (rituximab, ibritumomab, tiuxetan, and tositumomab), CD33 (gemtuzumab), CD52 (alemtuzumab), HER2/neu (trastuzumab), and EGFR (cetuximab, panitumumab) [14,17].

Among possible molecular targets, PD-L1 (Programmed death-ligand 1), a transmembrane protein highly expressed in several cancer cell types, is particularly interesting [18,19]. PD-L1 binds PD-1 (Programmed cell death protein 1), a transmembrane glycoprotein of the immunoglobulin superfamily expressed in T cells and pro-B cells [20]. The interaction of PD-1 and PD-L1 is an immune checkpoint that negatively regulates immune responses and favors tumor growth, and is particularly responsible for drug resistance [21]. Therefore, PD-1/PD-L1 interaction is now considered one of the main targets of modern oncology [22] and has been extensively investigated to identify cancer therapeutics [23,24]. Also, this approach has recently led to some compounds with potential anti-cancer activity, including D-peptide antagonists [25], peptides [26], cyclic peptides [27], and antibodies [28,29], either binding PD-1 or PD-L1.

In this work, we propose the use of a photoactive conjugate that consists of the clinical grade PD-L1 binding monoclonal antibody Atezolizumab [30], covalently linked to the well-known photosensitizer eosin. Photoactive conjugates containing eosin have been recently demonstrated to be effective against bacteria [31,32,33] and cancer cells [34]. The use of this targeting carrier is expected to show a synergic action through photo-induced cancer cell death and the limitation of the mechanisms of tumor cell immune evasion by blocking PD-1/PD-L1 interaction.

Atezolizumab, with durvalumab and avelumab, is one of the three anti-PD-L1 monoclonal antibodies approved by the FDA and is employed in the treatment of urothelial carcinoma, non-small-cell lung cancer (NSCLC), small-cell lung cancer, triple-negative breast cancer, hepatocellular carcinoma, and melanoma [35]. It has been applied in photo-immunotherapy only in one case so far, in combination with a different photosensitizer (Chlorin e6) on HCT-116, a colon cancer cellular line, demonstrating a decrease in the tumor mass larger than 50% in a mouse model upon illumination in comparison to dark conditions [36]. Moreover, it has been used in the intracellular light-controlled drug delivery method termed photochemical internalization, an innovative procedure to release a drug caged in endo/lysosomal compartments, exploiting the oxidative action of a photosensitizer (TPCS_2a_/fimaporfin) upon irradiation [37].

Using time-resolved and steady-state optical spectroscopies, we showed that the PS also retains its photoactivity when bound in the supramolecular construct. We then explored the potential of the photoactive complex between eosin and atezolizumab for the photo-immunotherapy of PD-L1-expressing NSCLC cell lines, and evaluated the relation between the photodynamic treatment outcome and the level of PD-L1 expression. Widefield and fluorescence microscopy, and direct stochastic optical reconstruction microscopy (dSTORM) were employed to assess the binding of Atezolizumab to the selected cultured NSCLC cell lines (H322 and A549). The PD-L1 expression level was determined using flow cytometry and dSTORM microscopy. Photoinactivation studies on investigated cell lines were performed, and the correlation between efficacy and PD-L1 expression level was discussed. Therefore, in this work, we show that (1) the superficial PD-L1 density on cancer cells is not the unique determining factor for the efficacy of a photodynamic treatment against this molecular target, and that (2) single-molecule localization microscopy can be efficiently used to quantify the density of PD-L1 receptors exposed on the cell membrane.

## 2. Materials and Methods

### 2.1. Chemicals

CD274 (PD-L1, B7-H1) Antibody (Catalog #13-5983-82) (human anti-PD-L1 primary antibody), AlexaFluor647-Mouse IgG1 Cross-Adsorbed Secondary Antibody (Catalog #A-21240), and AlexaFluor647 NHS ester were purchased from Thermo Fisher Scientific (Waltham, MA, USA). Eosin-5-isothiocyanate (EITC) was purchased from Sigma-Aldrich (St. Louis, MO, USA). Therapeutic antibody Atezolizumab was obtained from the inpatient pharmacy of the University Hospital of Parma.

The Atezolizumab extinction coefficient at 280 nm was evaluated from the amino acid sequence of the protein [38] and is estimated to be equal to 232,400 cm^−1^ M^−1^. The EITC and AlexaFluor647 molar extinction coefficients are tabulated as follows: ε (526 nm) = 95,000 cm^−1^ M^−1^ and ε (650 nm) = 270,000 cm^−1^ M^−1^, respectively. Then, the concentration of total antibody in conjugates was calculated from its absorbance at 280 nm after correcting for the dye absorbance at this wavelength.

### 2.2. Purification of Atezolizumab and Labeling with EITC or AlexaFluor647

Antibody purification was achieved by using a Sephadex G25, PD-10 column (Cytiva, Marlborough, MA, USA) to isolate the antibody from excipients. Atezolizumab was labeled with either EITC (EITC–Atezolizumab) for photoinactivation tests, or an N-hydroxysuccinidimidyl ester (NHS) derivative of AlexaFluor647 (Alexa647–Atezolizumab) for wide-field and single-molecule localization imaging. Both dyes react with the amine groups of lysine residues of the protein, to form stable conjugates. A concentrated stock solution of dye in DMSO buffer was added to a stock solution of antibody in PBS buffer at pH = 7, so that the final molar ratio antibody/dye was 1:6. The solution was incubated overnight at room temperature and then purified using a PD-10 column equilibrated with PBS buffer at pH = 7. The degree of labeling (DOL) of the antibody was determined spectroscopically using a Jasco V650 spectrophotometer (Jasco Europe S.R.L., Milano, Italy). The DOL indicates the average number of dye molecules coupled to each antibody molecule, and it is expressed as [EITC]/[Atezolizumab] molar ratio. An average number of 2 dye molecules per protein was obtained.

### 2.3. Spectral Properties of the Conjugate and ^1^O_2_ Production

Absorption spectra were acquired using the Jasco V-650 (Jasco Europe S.R.L., Milano, Italy) double-beam spectrophotometer. Steady-state fluorescence excitation, emission, and anisotropy spectra were recorded using the FS5 Spectrofluorometer (Edinburgh Instruments Ltd., Livingston, UK). Fluorescence decays were collected with the FLS920 time-correlated single-photon counting system (TCSPC) (Edinburgh Instruments Ltd., Livingston, UK), with pulsed LED excitation set at 500 nm, operated at a 5 MHz repetition rate. Lifetime decays were analyzed using a re-convolution mode for the fitting which operates the re-convolution between the exponential model and an acquired instrument response function (IRF). The quality of the fit was evaluated by minimizing the reduced chi-squared function and by a visual inspection of the weighted residuals and their autocorrelation.

The fluorescence quantum yield of the conjugate was evaluated with a comparative method [39], using eosin in PBS as a reference (Φ*_F_* = 0.24) [40]. All experiments were performed at 20 °C.

The decay of the triplet state of the EITC–Atezolizumab complex was followed at 522 nm upon excitation with the second harmonic of a pulsed Nd:Yag laser (Surelite I-10, Continuum, San Jose, CA, USA), using a previously described set-up [41]. The triplet quantum yield of EITC–Atezolizumab was determined using a comparative method, defined by the equation:

(1) $$\Phi_{T,X}=\Phi_{T,R}\left(\frac{{∆A}_{X}}{{∆A}_{R}}\right)\left(\frac{1-10^{-A_{R}}}{1-10^{-A_{X}}}\right)$$

where Φ*_T,R_* and Φ*_T,X_* are the triplet quantum yield of the reference compound and the conjugate, respectively, *A_R_* and *A*_X_ are the absorbance values at 532 nm, and Δ*A_R_* and Δ*A_X_* are the values retrieved from the experimentally measured transient absorbance changes. EITC was used as a reference, and the kinetics was followed at 526 nm for EITC–Atezolizumab and 524 nm for EITC, where bleaching of the ground state was observed.

In order to determine the singlet oxygen (^1^O_2_) quantum yield of EITC–Atezolizumab, we used a comparative method, with Rose Bengal as a reference and singlet oxygen sensor green (SOSG) (Invitrogen, Waltham, MA, USA). SOSG was dissolved in methanol to obtain a 5 mM stock solution, that was diluted to a final concentration of 1.5 μM in solutions containing Rose Bengal and the conjugate. The comparative method exploits the stoichiometric relationship between SOGS fluorescence emission intensity and the concentration of ^1^O_2_ produced.

The sample, the reference, and a control solution containing only SOGS at the same concentration were progressively illuminated using a frequency-doubled cw Nd:YAG laser (Laser Quantum, Manchester, UK, 1 W at 532 nm), attenuated to 30 mW. After each 20 s irradiation, up to a total of 160 s, the SOSG fluorescence emission was followed (λ_ex_ = 488 nm). The increase in emission intensity can be analyzed using a linear fitting and the slopes of the sample and the reference, corrected for the SOGS absorption, allow to determine the quantum yield of ^1^O_2_ according to the equation:

(2) $$\Phi_{\Delta ,X}=\Phi_{\Delta ,R}\left(\frac{{slope}_{X}}{{slope}_{R}}\right)\left(\frac{1-10^{-A_{R}}}{1-10^{-A_{X}}}\right)$$

where Φ_Δ,*R*_ and Φ_Δ,*X*_ are the ^1^O_2_ quantum yield of the reference compound (Rose Bengal) and the conjugate, respectively, *A_R_* and *A_X_* are the absorbance values at 532 nm, and *slope_R_* and *slope_X_* are the values retrieved from the linear fitting.

### 2.4. Cell Culture

H322 and A549 Human NSCLC cells were purchased from the American Type Culture Collection (ATCC, Manassas, VA, USA). ATCC authenticates the phenotypes of these cell lines on a regular basis. Human dermal fibroblasts (HuDe) were obtained from Istituto Zooprofilattico Sperimentale (Brescia, Italy).

H322 and A549 cells were cultured in Roswell Park Memorial Institute medium (RPMI 1640) supplemented with 2 mM Glutamine, 10% Fetal Bovine Serum (FBS), and 100 U/mL Penicillin-100 µg/mL Streptomycin. HuDe fibroblasts were cultured in Dulbecco’s Modified Eagle Medium (DMEM) supplemented with 2 mM Glutamine, 10% FBS, and 100 U/mL Penicillin-100 µg/mL Streptomycin. All cell models were maintained at 37 °C in a water-saturated atmosphere of 5% CO_2_ in air.

### 2.5. Evaluation of PD-L1 Expression

#### 2.5.1. Flow Cytometry

For the determination of PD-L1 membrane expression, phycoerythrin (PE) isotype control mouse IgG1κ (clone MOPC-21, Catalog #554680) and PE anti-human PD-L1 (clone MIH1, Catalog #557924) (BD Biosciences, San Jose, CA, USA) were used.

Briefly, after harvesting and washing with PBS, 5 × 10^5^ cells were incubated with PE isotype or PE anti-PD-L1 antibody in PBS + 0.5% Bovine Serum Albumin (BSA) at 4 °C in the dark for 30 min. Then, the cells were washed with cold PBS + 0.5% BSA and resuspended in this buffer for subsequent analysis.

Data acquisition was performed using a CytoFLEX Flow Cytometer (Beckman Coulter Life Sciences, Indianapolis, IN, USA); data analysis was carried out with Beckman Coulter’s Kaluza software (version 2.2).

#### 2.5.2. Microscope Setup

dSTORM images were acquired with an Oxford Nanoimaging (ONI) Nanoimager-S Mark III microscope. The microscope is equipped with a 100×/NA1.4 oil immersion objective (Olympus, Tokyo, Japan) and four excitation lasers (405, 488, 561, and 640 nm). The sample was irradiated using a total internal reflection configuration. The fluorescence emission was collected by the objective, split into two spectrally separated channels (576–620 nm and 665–705 nm) by a 640LP dichroic beam-splitter, and recorded using a CMOS camera (Hamamatsu OrcaFlash 4.0) to obtain 428 × 684 images with a pixel size of 117 nm.

#### 2.5.3. Sample Preparation and Acquisition for dSTORM Imaging

The cells (H322, A549, HuDe) were seeded in 8-well Chamber Slides (Thermo Scientific, Waltham, MA, USA, Nunc Lab-Tek II Chamber Slide) at 15 × 10^3^ cells per well. After two days, they were chemically fixed with 2% PFA (pH = 7.4) in PBS at room temperature for 15 min, and then rinsed three times with PBS. The cells were blocked in PBS + 2% BSA at room temperature for 20 min and finally washed twice with PBS. For immunostaining, the fixed cells were incubated with 5 µg/mL anti-PD-L1 primary monoclonal antibody in PBS + 1% BSA at room temperature for 1 h. After washing three times with PBS, the cells were incubated with 5 µg/mL AlexaFluor647-conjugated secondary antibody in PBS + 1% BSA + 0.05% tween20 at room temperature in the dark for 1 h, and then extensively rinsed with PBS. In control experiments, the cells were incubated as above, without adding the human anti-PD-L1 primary antibody. Right before imaging, 200 μL of fresh imaging buffer (5% *w*/*v* glucose, 100 mM cysteamine, 0.5 mg/mL glucose oxidase, catalase 40 mg/mL in PBS buffer, pH = 7.4) was added to the well.

Cell samples were acquired for 20.000 frames with a 15 ms camera exposure time using a continuous 640 nm excitation (~327 mW). At least 11 cells were acquired for each cell model and at least 5 cells for each control experiment with secondary antibody only. 

For the analysis performed to define the signal of individual receptors (see below), the sample was prepared and imaged as previously described, but using a lower concentration of primary antibody (0.125 μg/mL). Under this condition, only a fraction of well-isolated PD-L1 was fluorescently labeled on the cell surface.

#### 2.5.4. dSTORM Image Analysis

The acquired frame series were processed with the NimOS software 1.19.4 from ONI. In each frame, isolated fluorescence spots, corresponding to a single emitting dye, were fitted with a 2-D Gaussian model to determine the center position of the spot (localization). The localizations obtained in the acquired frames were brought together to reconstruct the final dSTORM image. Localizations that were too weak (below 300 detected photons) or acquired during initial equilibration (first 1500 acquisition frames) were discarded.

The PD-L1 density was evaluated using a custom MatLab script which requires the dSTORM localizations (x-y coordinates) and the corresponding bright field image as inputs. Regions of interest were selected manually on the bright files image by drawing a polygonal area that matched the cell shape. The script then reported the localization density within each selected cell area. The localization density was then divided by the number of localizations per single PD-L1, to obtain an estimate of the receptor density within the selected cell area.

In order to establish the mean number of localizations per single PD-L1 in our acquisitions, we used dSTORM images acquired at low concentrations of the primary antibody. A previously developed mean-shift algorithm [42,43] was used to identify localization clusters that reasonably correspond to a single receptor. The clustering bandwidth was set to 30 nm, which approximately corresponds to the dSTORM precision. Clusters with fewer than 5 localizations and with unreasonably large size (>100 nm) were discarded, yielding ~200 identified clusters per cell. The clusters were binned according to localization number, obtaining a non-symmetrical distribution that was fitted with a mono-exponential decay function to extrapolate the mean number of localizations per receptor, which is 14.3 [42] (Figure S1).

### 2.6. Photodynamic Inactivation of Cultured Cells

Cells were seeded at a density of 5 × 10^3^ cells per well in poly-L-lysine-coated 96-well microplates (Thermo Fisher Scientific, Waltham, MA, USA).

After two days, the EITC–Atezolizumab complex was administered at different concentrations (10 μM, 5 μM, and 1 μM) in phenol red-free FCS free RPMI 1640 medium, completed with 2% BSA, and the cells were incubated for 2 h at 37 °C in the dark. Cells incubated with antibody alone (at the highest concentration) and cells treated with EITC–Atezolizumab but not irradiated were used as controls.

The irradiation of cell cultures was conducted using a RGB LED light source (Studio par 64 CAN RGBWA+UV 12, Cameo, Anspach, Germany), whose green (523/40 nm) and blue (458/25 nm) output was selected. The irradiance at the surface of the 96-well plate was homogeneous and corresponded to 14.4 mW/cm^2^. The cells were irradiated for 60 min (which corresponds to a light fluence of 51.8 J/cm^2^) and then maintained in a humidified atmosphere with 5% CO_2_ at 37 °C, without light exposure, until analysis.

#### 2.6.1. Analysis of Cell Viability and Cell Death

Cell viability was evaluated using MTT and ATP assays.

MTT was added to cell cultures at the concentration of 1 mg/mL. After 1 h of incubation, the produced formazan crystals were solubilized by dimethyl sulfoxide (DMSO), and the absorbance was measured at 565 nm using a microplate plate reader (Infinite 200 PRO, TECAN, Männedorf, Switzerland).

The ATP assay was performed using the CellTiter-Glo 2.0 Assay (Promega Biosciences, San Luis Obispo, CA, USA), following the manufacturer’s instructions. The luminescent signals were recorded with a microplate plate reader (Infinite F200, TECAN).

Cell death was assessed via fluorescence microscopy after staining with Hoechst 33342 and Propidium Iodide (PI), as previously described [44].

#### 2.6.2. ROS Detection

Reactive oxygen species (ROS) production was assessed by using the ROS-Glo H_2_O_2_ assay according to the manufacturer’s instructions (Promega Biosciences). The H_2_O_2_ substrate dilution buffer (125 µM) was added immediately after completing PDT treatment, and the cells were incubated for 3 h at 37 °C. The assay was performed in non-lytic cell-based mode, which means that at the end of the incubation, 50 µL of medium containing the H_2_O_2_ substrate was transferred to a separate white plate and then 50 µL of ROS-Glo detection solution was added to make up the final volume of 100 µL in each well. The luminescence signals, proportional to the amount of H_2_O_2_ in the sample wells, were recorded with a microplate plate reader (Infinite F200, TECAN, Männedorf, Switzerland).

#### 2.6.3. Statistical Analyses

Statistical analyses were carried out using Matlab R2023a (The MathWorks, Inc., Natick, MA, USA). The statistical significance of differences among data was estimated using Student’s *t*-test. *p* values less than 0.05 were considered significant. Further details can be found in the figure captions, where data are expressed as means ± SD, where SD is the standard deviation, or means ± SEM, where SEM is the standard error of the mean.

## 3. Results and Discussion

### 3.1. Quantitative Determination of PD-L1 Expression Levels

#### 3.1.1. PD-L1 Receptor Density via Flow Cytometry

Clinical applications of photo-immunotherapy require the knowledge of the density of receptors chosen for targeting. Along with immunohistochemistry, flow cytometry is a standard technique to detect labelled receptors on cell membranes and compare their expression between different types of cells.

**Figure 1:**
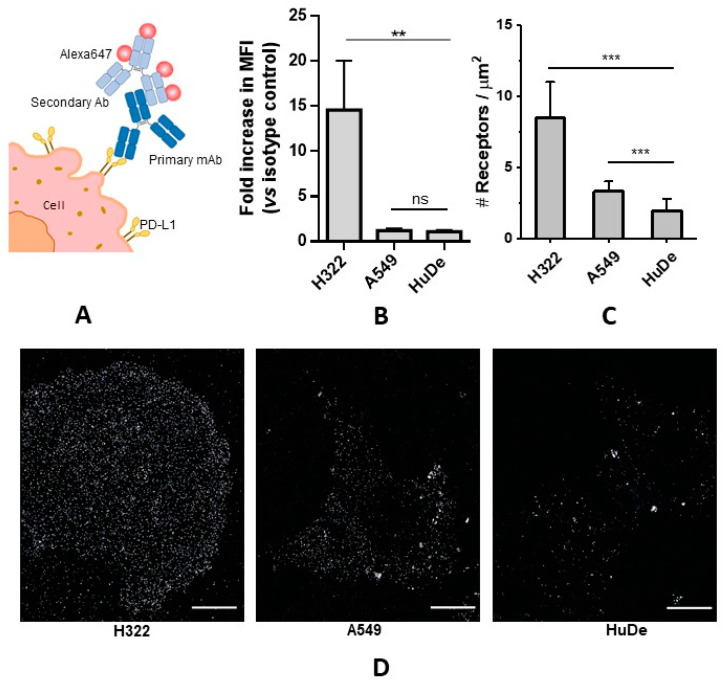
Flow cytometry and dSTORM analysis of PD-L1 cell surface expression. (**A**) Cartoon representing the PD-L1 immunostaining method used for H322, A549, and HuDe cells, using a primary PD-L1 antibody (mAb) and AlexaFluor647-conjugated secondary antibody (Ab). (**B**) Flow cytometry analysis of PD-L1 expression levels. After harvesting, the H322, A549, and Hude cells were stained with isotype control or PE-PD-L1 antibody and analyzed. For each cell model, PD-L1 surface expression levels are expressed as a fold increase of median fluorescence intensity (MFI) versus the corresponding isotype control, and are mean values ± SD of at least three independent experiments. ** *p* < 0.01, ns not significant vs. HuDe cells. (**C**) Quantification of PD-L1 membrane receptors per μm^2^ using dSTORM. For each cell model, a minimum of 10 cells were analyzed. *** *p* < 0.001 vs. HuDe cells. (**D**) Representative dSTORM images of PD-L1 membrane receptors on H322, A549, and HuDe cells. The fixed cells were incubated with 5 µg/mL anti-PD-L1 primary monoclonal antibody at room temperature for 1 h, and after washing three times with PBS, they were incubated with 5 µg/mL AlexaFluor647-conjugated secondary antibody at room temperature in the dark for 1 h (details in Section 2.5.3). Scale bars: 10 μm.

Using flow cytometry, we investigated the PD-L1 expression levels in two different human primary lung adenocarcinoma cell lines previously reported to be low-level (A549) and high-level (H322) PD-L1-expressing [45]. Since PD-L1 may also be expressed in non-hematopoietic normal cells [46], at a very low level, human dermal fibroblasts (HuDe) were also considered. As shown in Figure 1B, H322 cells expressed significantly higher levels of PD-L1 (14 ± 5) in comparison with the other cell models, while no difference emerged between A549 (1.2 ± 0.2) and HuDe cells (1.1 ± 0.1). The reported values are expressed as a fold increase of median fluorescence intensity with respect to the corresponding isotype control. This result shows that the PD-L1 expression level in H322 seems to be approximately twelve times higher than in A549.

#### 3.1.2. PD-L1 Receptor Density via dSTORM

In order to validate the cytofluorimetric analysis and provide single-molecule detection and quantification of PD-L1 receptors exposed on the cell surface, super-resolution microscopy images were acquired using a dSTORM approach [47]. In dSTORM, dyes reversibly switch between fluorescent and dark states in response to specific buffer components and photo-excitation. During a time-course acquisition, only a subset of well-isolated emitting fluorophores is detected in every frame, whose position (named localization) is detected with nanoscale precision. All localizations collected in the acquisition frames reconstruct a final image, with nanoscale resolution.

For dSTORM imaging, the PD-L1 receptors were immunostained on fixed H322, A549, and HuDe cells, using a primary anti-PD-L1 monoclonal antibody and a secondary antibody, conjugated with AlexaFluor647 (Figure 1A). Figure 1D shows representative dSTORM images of PD-L1 exposed on the basal membrane of H322, A549, and HuDe cells. The chosen models qualitatively exhibit different levels of membrane PD-L1. Among them, H322 cells showed the highest PD-L1 level, while non-tumoral HuDe cells displayed a relatively low level. A549 cells qualitatively appeared to have a signal comparable—or slightly higher—than HuDe. Besides the qualitative comparison of cell models, we were interested in providing a quantification of the overall PD-L1 density on the basal membrane by using dSTORM imaging. In comparison to common bulk techniques for the investigation of receptor expression levels, which are typically limited to relative comparisons of different cell types, dSTORM is endowed with superior sensitivity, that enables the detection and counting of individual membrane protein receptors [48]. The histogram in Figure 1C represents the density of PD-L1 receptors, expressed as receptors per square µm, detected using dSTORM, compared to a control with secondary antibody only, that reports background signal, e.g., due to non-specific surface interactions. The absolute receptor number was estimated following a previously established method [42], to obtain the number of localizations detected for a single PD-L1. This is necessary because one antibody used for immunostaining bears multiple dyes and, additionally, a single fluorophore generates multiple localizations in an acquisition, originated by several switching events.

The PD-L1 density of the H322 cell line was found to be (8.5 ± 2.5) PD-L1/µm^2^ (mean ± SD), which confirmed H322 to be the model with the highest expression among those studied, while the A549 and HuDe densities were (3.3 ± 0.6) and (2.0 ± 0.8) PD-L1/µm^2^, respectively. Large errors reflect the intrinsic cell-to-cell heterogeneity of the biological sample. Moreover, A549 cells exhibited a statistically significant increase in receptor counting over the control, obtained with secondary antibody only, while there was no difference between the HuDe sample and its control, indicating that PD-L1 levels on this cell model are too low to be detected over the background, at least with this method. Our results are in line with the findings of a previous study that followed a similar approach to estimate CD19 expression levels on myeloma cells [48].

It is worth noticing that this approach brought out a statistically significant difference between A549 cells and HuDe cells that was not appreciable in flow cytometry analysis, as a consequence of the higher single-molecule sensitivity of dSTORM. 

From the receptor density values, we roughly estimated that ~8500 PD-L1 copies were found on the basal membrane of H322 cells, while ~2500 were found on A549 cells. These absolute numbers are affected by relatively large uncertainties, e.g., due to the variable receptor-dye stoichiometry, the residual non-specific background signal, the possibly incomplete receptor labeling on the basal membrane, and the intrinsic imperfections of analysis. Still, they can be regarded as a useful absolute parameter for interpreting the efficacy of delivery systems using a quantitative approach. 

Finally, the partial discrepancy observed between flow cytometry and dSTORM (Figure 1B,C) in determining the relative difference of PD-L1 levels between cell models, can be explained by inherent technical differences in terms of sample preparation, acquisition, and sensitivity, together with a possibly incomplete immunolabeling of PD-L1 on the basal membrane [49].

As a final remark, we note that super-resolution microscopy offers several important advantages over flow cytometry, overcoming its limitation in determining ultra low expression levels of antigens [48], and being able to report on other membrane-related properties such as the distribution of glycans [50] and protein organization on plasma membranes [51].

### 3.2. EITC–Atezolizumab Complex for Photo-Immunotherapy

**Figure 2:**
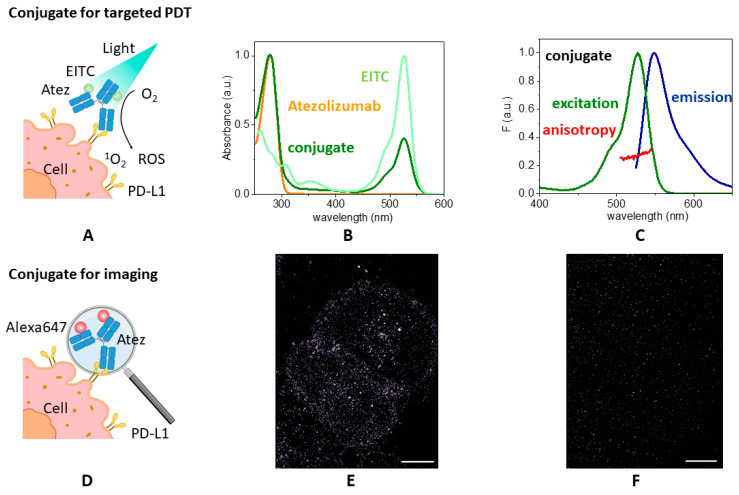
Conjugation of EITC to Atezolizumab. (**A**) Cartoon representing the supramolecular complex formed by EITC and Atezolizumab (Atez) used for the photodynamic inactivation of cancer cells. (**B**) Normalized absorption spectra of Atezolizumab in PBS buffer at pH = 7 (orange), EITC in PBS buffer (light green), and the complex EITC–Atezolizumab in PBS buffer at pH = 7 (green). The absorption spectra were normalized at 280 nm (Atezolizumab and conjugate), or 525 nm (EITC in PBS). (**C**) Normalized fluorescence excitation (green, peak at 527 nm, emission collected at 550 nm) and emission (blue, peak at 549 nm, excitation at 520 nm) for EITC–Atezolizumab in PBS buffer at pH = 7. Fluorescence excitation anisotropy is reported in red (emission collected at 550 nm). (**D**) Cartoon representing the supramolecular complex formed by AlexaFluor647 and Atezolizumab (Atez) used for visualizing the binding of Atezolizumab to cancer cells. (**E**) Representative dSTORM image of PD-L1 membrane receptors immunostained with Atezolizumab directly conjugated to AlexaFluor647 on H322 cells. (**F**) Corresponding control dSTORM image obtained using an AlexaFluor647-labelled IgG. Scale bars: 10 μm.

In order to evaluate if cells that express a higher level of PD-L1 are more sensitive to photo-immunotherapy than cells with a lower level, we realized a nanostructured photo-active construct, based on Atezolizumab, sketched in Figure 2A, and assessed its binding capability to cell lines expressing PD-L1 to a different level.

#### 3.2.1. Spectral Properties of EITC–Atezolizumab

Eosin 5-isothiocyanate (EITC) was covalently conjugated to Atezolizumab, exploiting the reaction with lysine residues. EITC shows an absorption band at 524 nm in PBS buffer, that shifts to 525 nm when bound to Atezolizumab. The concentration of the conjugate was determined from the absorbance at 280 nm, introducing a correction for the absorption of EITC at this wavelength (Figure 2B). The degree of labeling (DOL), indicated as [EITC]/[protein], can be calculated using the molar extinction coefficients of the protein and EITC, and represents the average number of PS molecules conjugated to the antibody. In our preparation, the DOL was approximately 2. As already observed for EITC bound to streptavidin [31], the emission properties of EITC are not dramatically affected by conjugation at low DOL. The fluorescence excitation spectrum closely resembles the absorption spectrum of EITC, and no substantial changes in fluorescence emission spectra appear after the binding (Figure 2C). Moreover, using eosin in PBS as a reference (Φ*_F_* = 0.24 [40]), we estimated the fluorescence emission quantum yield of the EITC–Atezolizumab complex as 0.11, smaller than the corresponding value for EITC in PBS, for which Φ*_F_* = 0.18 [31]. DOL is most likely too low to explain the observed quenching in terms of the interaction and aggregation between fluorophores. We suggest that quenching by amino acids of the antibody may contribute to decrease Φ*_F_* and Φ*_T_* (vide infra).

The formation of a stable complex between the PS and the antibody is proved by steady-state fluorescence anisotropy and fluorescence lifetime measurements. In fact, the anisotropy of EITC in PBS is negligible, whereas the anisotropy of the conjugate varies from 0.25 to 0.31 between 500 and 550 nm, reflecting a reduction in the rotational mobility of the PS within its excited state lifetime, thus indicating a successful conjugation. According to these results, the fluorescence lifetime of EITC linked to Atezolizumab is described by a bi-exponential decay with a mean lifetime τ = 1.31 ± 0.03 ns, slightly longer than the single lifetime associated with free eosin in solution, equal to τ = 1.21 ns [40].

#### 3.2.2. Singlet Oxygen Production by EITC–Atezolizumab

The photosensitizing properties of EITC are related to the significant probability of this molecule to undergo an intersystem crossing process, that populates the triplet state with quantum yield Φ*_T_* = 0.49 [31]. Following the time evolution of the ground state bleaching, we estimated that when EITC is bound to Atezolizumab, the triplet quantum yield decreases to 0.20 ± 0.05 (Equation (1)). This suggests that EITC is probably bound in a region of the protein rich in amino acids able to quench the triplet state of the PS, as observed, for example, for the EITC–streptavidin complex, for which Φ*_T_* becomes 0.1 [31].

We further investigated the capability of the EITC–Atezolizumab complex to generate singlet oxygen. Using a comparative method based on SOSG, we determined the quantum yield of the photo-active conjugate, according to Equation (2). Using Rose Bengal as a reference (Φ_Δ_ = 0.76 [52]), we obtained the value Φ_Δ_ = 0.11 ± 0.02. The singlet oxygen quantum yield is lower than the one observed for free eosin, for which Φ_Δ_ exceeds 0.5 [53]. It can be hypothesized that the antibody shields the PS from oxygen to some extent, although not enough to prevent the formation of ^1^O_2_, as we previously observed in other protein-based photoactive constructs [54]. Finally, ^1^O_2_ quenching by amino acids of the antibody may contribute to the lower Φ_Δ_ value in comparison to Φ*_T_*.

#### 3.2.3. Binding Capability of the Conjugate to Cell Lines Expressing PD-L1

In order to confirm the ability of the EITC–Atezolizumab complex to bind PD-L1, we performed fluorescence microscopy experiments. As shown in Figure 2C, the photosensitizer retains fluorescence emission when conjugated to Atezolizumab, but shows a relatively low fluorescence quantum yield. For imaging purposes, as schematically sketched in Figure 2D, EITC was thus replaced by a brighter dye (AlexaFluor647), allowing the localization of the fluorophore–Atezolizumab conjugated at the single-molecule level with dSTORM imaging.

To provide an effective membrane staining, labelled Atezolizumab (1 μM) was added to living H322 cells, and after 1 h of incubation the cells were fixed with 2% PFA, as previously described. Stained PD-L1 receptors were finally visualized by excitation of AlexaFluor647 at 640 nm. Figure 2E shows dSTORM images of AlexaFluor647–Atezolizumab on H322 cells, collected in a TIRF configuration to provide an ultrathin section of the basal membrane, and confirms the binding capability of the conjugate. Moreover, the specificity of the binding was proved by incubating cells with a non-specific control AlexaFluor647-labelled IgG. No significant interaction was detected (Figure 2F). Similar results were obtained for A549 cells.

### 3.3. Photoinactivation Treatment with the Conjugate

To optimize the culture conditions required for photodynamic treatments and assess the efficacy of the procedure, we firstly used hypericin, a naturally occurring photosensitizer, whose photodynamic activity is widely recognized and has also been proven by our group in other cancer cell models in vitro [55].

In addition to the MTT cell viability assay conducted 24 h after cell irradiation, we used a luminescent assay that measures the level of H_2_O_2_ as a suitable method to evaluate the photosensitizer-mediated production of ROS in irradiated cells. In fact, a variety of evidence indicates that H_2_O_2_ is one of the most relevant species contributing to ROS accumulation under photodynamic treatment, besides singlet oxygen [56]. As expected, hypericin induced a significant increase in ROS levels (Figure S2A), which resulted in a strong cytotoxic effect confirmed via MTT assay (Figure S2B).

Based on these results, the same protocol was used for the subsequent experiments, aimed at evaluating the effects of the EITC–Atezolizumab conjugate. In particular, in order to determine dose-dependent PDT effects of the EITC–Atezolizumab complex on the viability of cancer cells, experiments maintaining constant the duration of incubation and illumination, and varying the concentration of the administrated conjugate were performed.

Preliminary tests using sub-micromolar concentrations resulted in no significant effects and are not reported. This is in line with previously published works, where Atezolizumab [36,37] was used in definitely larger concentrations than the dissociation constant of the antibody, 0.19 nM and 0.62 nM for the dimeric and monomeric form of PD-L1, respectively [35].

**Figure 3:**
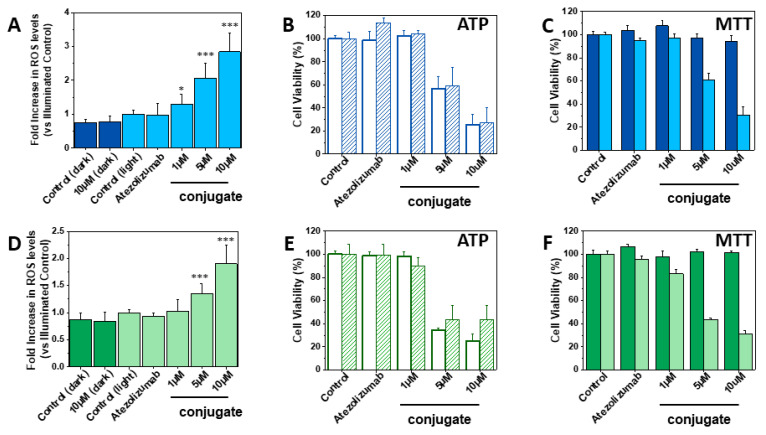
ROS-induced oxidative stress and cell viability for H322 (blue) and A549 (green) cells after PDT treatment with the EITC–Atezolizumab conjugate. The cells were treated with increasing concentrations of the EITC–Atezolizumab conjugate or Atezolizumab alone and exposed to the green and blue light output (14.4 mW/cm^2^) from a LED lamp for 60 min (corresponding to a light fluence of 51.8 J/cm^2^). (**A**,**D**) ROS production evaluated 3 h after light exposure. Bars represent the increase in ROS levels over control cells exposed to light only. Untreated cells and cells treated with the EITC–Atezolizumab conjugate at the highest concentration but not exposed to light were used as additional controls (dark blue and dark green bars). Data are the means ± SD of three independent experiments performed in triplicate (* *p* < 0.05, *** *p* < 0.001 versus illuminated control). (**B**,**E**) ATP assay for cell viability performed 3 h (white bars) and 24 h (dashed bars) after irradiation. Relative viabilities are calculated as a percentage of control. Data are the means ± SEM of three (H322) or two (A549) independent experiments with four replicates. (**C**,**F**) Effect of PDT treatment on cell viability evaluated via MTT assay at 24 h. Histograms are representative of at least three independent experiments with five replicates carried out in parallel under light and darkness conditions (light blue/light green bars and dark blue/dark green bars, respectively). Cell viability is calculated as a percentage of control and is expressed as the mean ± SD.

As reported in Figure 3A, H322 cells treated with increasing concentrations of the conjugate (1, 5, and 10 μM) and exposed to light for one hour produced greater amounts of ROS than control cells or cells treated with Atezolizumab alone, with an effect more pronounced at the highest concentration used. Of note, neither illumination per se nor the conjugate in non-irradiated cells had effects on ROS production.

Then, we investigated the impact of the EITC–Atezolizumab conjugate on cell viability and, in order to detect possible early effects, we used the ATP assay due to its higher sensitivity [57], in addition to the MTT assay.

When the conjugate was used at 1 µM in H322 cells, cell viability was not affected. In contrast, the ROS-mediated oxidative stress induced by the conjugate at 5 and 10 µM concentrations led to a significant reduction of cell viability by ~40% and 75%, respectively, an effect already detectable 3 h after light exposure and maintained even after 24 h, as indicated by the ATP assay (Figure 3B). The MTT assay (Figure 3C) confirmed the level of photo-killing of cells and also demonstrated that irradiation was ineffective in control cells or cells treated with Atezolizumab alone; in addition, even the cells treated with the EITC–Atezolizumab conjugate without irradiation remained viable, indicating that cell toxicity is specifically associated with the photodynamic activity of the conjugate.

A quantitative analysis of cell death was performed via fluorescence microscopy after staining with Hoechst33342 vital dye and with propidium iodide (PI), which enters only non-viable cells that have lost their membrane integrity. Twenty-four hours after the photodynamic treatment with the conjugate at 10 µM, 50% of PI-positive dead cells were detected (Figure S3), in good agreement with the evaluation by the other assays.

In addition, no early apoptotic nuclei excluding PI were observed during this analysis, and the morphological alterations of PI-positive nuclei resemble those more typical of necrosis than apoptosis.

The type of cell death induced by PDT not only depends on the PDT dosage (i.e., photosensitizer concentrations and light dose), but is also influenced by the localization of the photosensitizer. Photosensitizers targeting plasma membrane receptors generally cause necrosis, inducing swelling and plasma membrane rupture [58]. Recently, a type of regulated necrosis, with features reminiscent of ferroptosis, has been described in cells undergoing ROS-mediated lipid peroxidation after photodynamic treatment with intermediate levels of hypericin [59]. Besides this work, there is an increasing number of studies in recent years suggesting that ferroptosis may play a role in photodynamic treatments; interestingly, most of them employ third-generation PSs, including PSs conjugated with monoclonal antibodies [60]. We did not proceed with further investigation to clarify the type of death induced by the treatment with the EITC–Atezolizumab conjugate, but we cannot exclude the involvement of ferroptosis, which usually show necrosis-like morphological changes including moderate chromatin condensation and the loss of plasma membrane integrity [61].

In A549 cells, the conjugate at 1 µM had no effect on cell viability, being unable to trigger ROS production (Figure 3D). However, higher concentrations of the conjugate (5 and 10 μM) induced a rapid and substantial decrease of cell viability also in this cell model, even if the amount of ROS generated was lower than that measured in H322 cells under the same conditions. The ATP assay performed 3 and 24 h after illumination (Figure 3E) and the MTT assay (Figure 3F) suggested that the photoinactivation was a little more efficient in A549 than in H322 cells when the conjugate was used at 5 µM, while the effect was comparable between the two models at 10 µM, within the error bars. Similar results were reported by Pan et al. using a Chlorin-e6-functionalized Atezolizumab at the same concentrations on HCT-116 cells [36].

These results overall demonstrate that the EITC–Atezolizumab complex is effective in both H322 and A549 cells regardless of PD-L1 expression levels. This suggests that the intrinsic features of the cells, which may include the sensitivity to ROS-mediated oxidative stress, may play a relevant role in the response to photodynamic treatments and should be taken into account when evaluating the efficacy of targeted PDT. It is evident that this is only a pre-clinical study in vitro; therefore, it will be important to investigate if these cellular effects are confirmed also in vivo, but this evaluation goes beyond the aims of this work. Nevertheless, it is worth noting that the clinical outcomes of immunotherapy with PD-1/PD-L1 checkpoint inhibitors including Atezolizumab have not been unequivocally correlated with PD-L1 expression, and tumor responses have been reported not only in patients with high PD-L1 expression but also in those with low PD-L1 levels [62].

Several studies reported similar findings with different receptors. For instance, in HER2-expressing cholangiocarcinoma cells, effectiveness does not seem to depend on receptor expression levels [63], whereas such correlation is evident in different HER2-expressing breast cancer cell lines [64]. Two different B-cell lymphoma cell lines were reported to express a similar amount of CD20, but showed very different responses to photodynamic treatments [65]. In the case of colorectal cancer cells, a higher CEA availability did not ensure a larger therapeutic effect [66]. Finally, while the expression level of EGFR was shown to be positively correlated to photo-immunotherapy efficacy in glioblastoma cells [67,68], contradictory results were reported for human head and neck squamous cancer cell lines [58,69], thus confirming that receptor expression levels do not appear to be the only decisive factor in photo-immunotherapy efficacy.

## 4. Conclusions

It is generally assumed that photo-immunotherapy efficacy depends on the cellular density of receptors, and that a high level of antigens expressed on tumor membranes is essential for the binding of antibodies that act as targeting agents [70]. This could impose restrictions on the use of this methodology, limiting its use to a reduced ensemble of patients. However, there is no agreement in the literature on this topic, as demonstrated by studies using both different conjugates and tumor lines.

In general, the evaluation of the level of antigens expressed on tumor plasma membrane is obtained through flow cytometry measurements, that allow only a relative comparison between different cellular lines. To overcome the limits of this qualitative analysis, we applied dSTORM to obtain a quantitative estimate of the PD-L1 density on the plasma membrane of two NSCLC tumor lines (H322 and A549) and a control cell model (HuDe).

We produced a supramolecular complex that is fully photoactive, binds the PD-L1 receptor with high affinity, and uses Atezolizumab, an antibody approved in clinical practice as a targeting species. We have shown that the photo-immunoconjugate can be specialized to obtain a photosensitizing compound, using Eosin-5-isothiocyanate as a photosensitizer, or turned into a fluorescent tag for detecting the target receptor, using AlexaFluor647 as a fluorophore.

We then applied the photo-immunoconjugate to target PD-L1 and investigated the effect of PDT on cell viability. While H322 and A549 cell viability was affected by the treatment, no correlation appears to exist between PDT efficacy and PD-L1 expression levels, an indication that mechanisms other than the simple receptor density determine the outcome of the treatment. This finding is in keeping with previous reports and brings additional evidence that case-by-case, detailed investigations are needed to evaluate the effect of PDT when receptors are targeted by photo-immunoconjugates. However, a detailed study of the role of intrinsic biological properties of tumor cell lines able to determine a different response to the PDT treatment, such as different death pathways or a different level of expression of other proteins, is beyond the aim of this work and will be the subject of future investigations. Although PD-L1 density does not seem to be the only factor that defines the cellular response to photo-immunotherapy, our results confirm that PD-L1-targeted PDT also represents a promising approach for low-PD-L1-expressing cells, as also observed by Sato and coworkers [70]. This demonstrates that a photo-immunotherapy approach that uses PD-L1 as the target, can provide relevant advantages. First of all, this immune checkpoint molecule is present in a wide variety of solid tumors [71], making the methodology potentially useful for a large number of patients. Second, some antibodies targeting PD-L1 are clinically approved by the FDA, so they represent promising candidates for targeted photo-immunotherapy. Third, as demonstrated in this work, the effectiveness of the treatment can also be relevant in the case of low-PD-L1-expressing cells, overcoming a limit of using antibodies that in general need a high level of antigen expression. Fourth, targeting PD-L1 on cancer cells allows us to block PD-1/PD-L1 interaction, limiting the mechanisms of tumor cell immune evasion, possibly reducing myeloid-derived suppressor cells in the tumor microenvironment [72,73] and favoring T cell activation.

Future experiments on PD-L1 knockout and overexpressing cellular lines will allow us to expand the PD-L1 density range and further explore the correlation between the receptor expression levels and the outcome of photo-immunotherapy treatment.

## Data Availability

The data presented in this study are available in this article (and Supplementary Materials).

## Supplementary Materials

The following supporting information can be downloaded at: https://www.mdpi.com/article/10.3390/pharmaceutics15122776/s1, Figure S1: Histogram representing the number of localizations per single PD-L1 receptor on H322 cells. Figure S2: Optimization of PDT protocol using hypericin. Figure S3: Assessment of cell death using fluorescence microscopy in H322 cells stained with Hoechst33342/PI 24 h after PDT treatment.
