# Supplementary material for: A Photoactive Supramolecular Complex Targeting PD-L1 Reveals a Weak Correlation between Photoactivation Efficiency and Receptor Expression Levels in Non-Small-Cell Lung Cancer Tumor Models
Source: Pharmaceutics. 2023 Dec 14;15(12):2776. doi: 10.3390/pharmaceutics15122776 (PMC10747218; doi:10.3390/pharmaceutics15122776)
Supplement: Supplementary file 1 [file pharmaceutics-15-02776-s001.zip › pharmaceutics-2720685-supplementary.pdf]

Supplementary Information

A photoactive supramolecular complex targeting PD-L1 reveals a weak correlation between photoactivation efficiency and receptor expression level in NSCLC tumor models

Pietro Delcanale [1], Manuela Maria Alampi [1**], Andrea Mussini[1], Claudia Fumarola [2], Maricla Galetti [3], Pier Giorgio Petronini [2], Cristiano Viappiani [1], Stefano Bruno [4], and Stefania Abbruzzetti [1,*]**

1 Department of Mathematical, Physical and Computer Sciences, University of Parma, 43124 Parma, Italy; pietro.delcanale@unipr.it (P.D.); manuelamaria.alampi@unipr.it (M.M.A.); andrea.mussini@unipr.it (A.M.); cristiano.viappiani@unipr.it (C.V.)
2 Department of Medicine and Surgery, University of Parma, 43125 Parma, Italy; claudia.fumarola@unipr.it (C.F.); piergiorgio.petronini@unipr.it (P.G.P.)
3 Department of Occupational and Environmental Medicine, Epidemiology and Hygiene, INAIL-Italian Workers' Compensation Authority, 00078 Rome, Italy; m.galetti@inail.it
4 Department of Food and Drug, University of Parma, 43124 Parma, Italy; stefano.bruno@unipr.it
* Correspondence: stefania.abbruzzetti@unipr.it
† These authors contributed equally to this work.

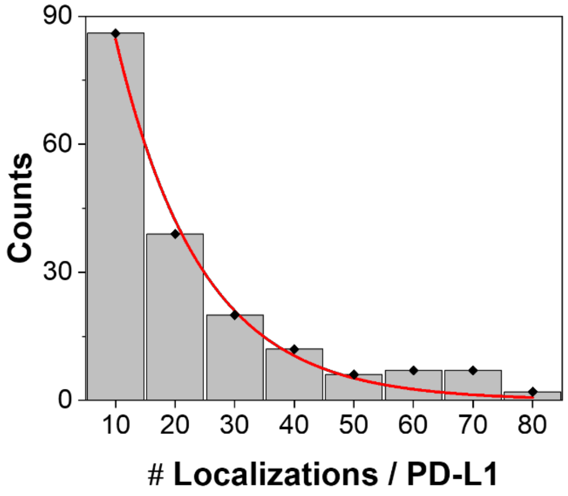

Figure S1. Histogram representing the number of localizations per single PD-L1 receptor on H322 cells stained with low concentration of primary monoclonal antibody. The histogram was fitted with a mono-exponential decay function to extrapolate the mean.

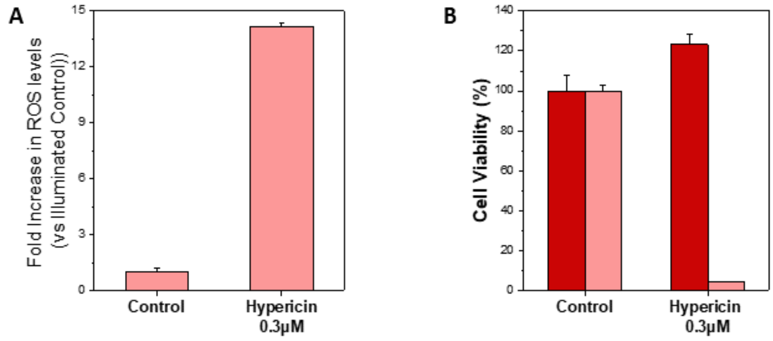

Figure S2. Optimization of PDT protocol using Hypericin. (A) ROS-mediated oxidative stress evaluated 3 hours after irradiation. Bars represent the increase of ROS levels over control cells exposed to light only. Data are the mean ± SD of two experiments performed in duplicate. (B) Cell viability at 24 hours assessed by MTT assay. Data is representative of two experiments carried out in parallel under light and darkness conditions (pink bars and red bars, respectively).

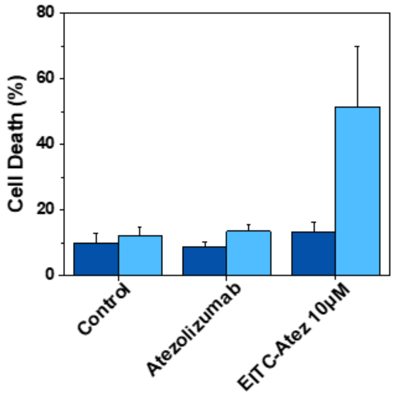

Figure S3. Assessment of cell death by fluorescence microscopy in H322 cells stained with Hoechst33342/PI 24 hours after PDT treatment. Percentages of cell death were calculated by determining the ratio of PI-stained cells to total cells per field. Ten fields per condition were counted. Graph represents the mean ± SD of at least three independent experiments carried out under darkness (dark blue) and light (light blue) conditions.
